# Al and Zr Porous Clay Heterostructures as Removal Agents of Basic Blue-41 Dye from an Artificially Polluted Solution: Regeneration Properties and Batch Design

**DOI:** 10.3390/ma14102528

**Published:** 2021-05-13

**Authors:** Hmoud Al Dmour, Fethi Kooli, Ahmed Mohmoud, Yan Liu, Saheed A. Popoola

**Affiliations:** 1 Department of Physics, Faculty of Science, Mu’tah University, Mu’tah 61710, Jordan; hmoud79@mutah.edu.jo; 2 Department of Chemistry, Faculty of Science, Islamic University of Madinah, Al-Madinah Al-Munawwarah 42351, Saudi Arabia; abiodun@iu.edu.sa; 3 Petroleum Technology, Operated Offshore Oil Field Development, Qatar Petroleum, Doha 3212, Qatar; caadil77@yahoo.co.uk; 4 Institute of Chemicals and Engineering Sciences, 1 Pesek Road, Jurong Island, Singapore 627833, Singapore; liu_yan@ices.a-star.edu.sg

**Keywords:** intercalated clays, porous clay heterostructures, Basic Blue-41, removal, regeneration, batch design

## Abstract

The removal of Basic Blue-41 dye molecules was carried out by using two doped porous clay heterostructures by aluminum (Al) or zirconium (Zr) species. The proposed method of synthesis showed its efficiency, starting from Al or Zr intercalated hydrolyzed species, prior to its reaction with dodecylamine (C_12_ amine) and tetraethyl orthosilicate (TEOS) as a silica source. The intercalated precursors and their porous clay heterostructures (PCH) derivatives were characterized by different techniques. Solid NMR technique proved the presence of Al species into the intercalated silica between the clay sheets, and in addition to Si in different environments within the PCH materials. The Zr-PCH material exhibited a higher surface area and pore volume compared to its Al-PCH counterpart, with a mesoporous character for both materials. A maximum removed amount of 279 and 332 mg/g was achieved and deduced from the Langmuir equation. The regeneration tests revealed that the removal efficiency of Zr-PCH was retained after five regeneration runs, with a loss of 15% of the original value; meanwhile, the Al-PCH lost 45% of its efficiency after only three cycles. A single-stage batch design was proposed based on the Langmuir isotherm parameters. The increase of the removal capacity of Zr-PCH led to the reduction of the required amounts for the target removal of BB-41 dye compared to Al-PCH.

## 1. Introduction

Humanity will face problems of freshwater supplies in near future, due to the increase of its demand in the daily life. Growing water scarcity is now one of the leading challenges facing the humanity due to population growth, migration, urbanization, and industrialization, in conjunction with increase in production and consumption. Among the sectors that consumed a huge amount of fresh water are the agricultural and industrial activities [1].

The dyes are hazardous once they are released into water streams, as they can considerably affect the photosynthetic activity in the aquatic environment; in addition, their degradation products, such as naphthoquinone, can be carcinogenic. Thus, their presence in drinking surface water can be harmful [2,3]. It is stated that there are more than 100,000 commercially available dyes, with a production over 7 × 10^5^ metric tons per year [4]. They were used in different industrial sectors, but mainly in the textiles industries, where about 9% of the total amount of dyestuffs produced in the world is discharged in textiles wastewaters [5]. The synthetic origin and complex aromatic structure of the dyes cause them to be more noncompliant to biodegradation [6,7]. Scientists and policymakers were beginning to take more warning of this vital problem to find an appropriate solution, and environmental legislation obligates industries to remove colors from their dye-containing effluents before disposal into bodies of water [5,6,7].

Up to our days, various decontamination techniques, including biological, chemical, and physical treatments, have been adopted to remove toxic compounds, especially synthetic organic dyes from environmental media in both industrial and municipal wastewaters [8,9,10,11]. These established processes have inherent limitations, such as design complexity and financial inputs, and thus it is necessary to look for efficient and simple methods. In this regard, the application of the adsorption strategy provided a simple, low cost, and relatively fast methodology with high efficiency. This conventional strategy is used in advanced wastewater treatment due to its ease of operation and flexibility in adsorbent design. This method also produces no harmful by-products and prevents sludge formation during the removal process. Numerous studies have been conducted and supported that adsorption emerges to the more efficient method for dye removal from aqueous discharges, due to its positive points such availability of raw and cheap absorbents, simple operation, the design, and insensitive of toxic substances [11].

In this regard, different materials were proposed and summarized in multiple reviews. Until now, various synthetic and natural adsorbents were used for removing organic dyes, which is discussed in some useful review reports [11,12,13,14]. Notably, developing adsorbents with improved characteristics to be used for the adsorption process is an ongoing trend. One of the candidates that attract a lot of attention are the clay minerals, they exhibited interesting properties with low cost, large availability, large quantity in nearly all continents of the world thermal, and chemical stability in a wide range of pH [15].

These removal properties of the clay minerals could be tuned by the modification of the starting materials by different means either physical or chemical ones, depending of the types of pollutants. The isomorphous substitution of Al^3+^ for Si^4+^ in the tetrahedral layer and Mg^2+^ for Al^3+^ in the octahedral layer creates a negative surface charge of clay minerals [16], and made difficult the removal of acidic dyes negatively charged, due to the electrostatic repulsion between the layers and the dyes. To overcome this problem, a modification of the host clay by organic cations such as alkylammonium and other organic cations have changed the properties of the resulting organoclays (OCs) and transformed them to good removal agents for these contaminants [17]. The quaternary alkyl ammonium cations are the favored surface modifiers due to their inexpensive and simpler treatment. [18]. The choice of the clay minerals and the structure of the surfactants allowed us to conceive the properties of the OCs. It has been reported that the structure, chemical characteristics of the clay mineral, and the counteranions of surfactants had a strong effect during the synthesis of the OCs [19,20]. In the case of positively charged molecules, such as basic dyes, the electrostatic attraction between the positive dye and the negative charged layer made it ready to remove these dyes. However, researchers were interested in improving the uptake amounts by modifying the starting clay minerals by several methods to improve their physicochemical properties, to increase the surface area of these materials, and the numbers of removal sites. The efficient acid activation method consists of reacting a solid clay mineral with a certain volume of inorganic or organic acid for a certain period of time and at different temperatures [21]. A mild acid treatment produces an increase of the porosity, so these solids were used as an absorbent of heavy metals and basic dyes [22,23].

The alkylammonium established organoclays exhibited a low regeneration capability due to their low thermal stability at temperatures above 200 °C [24]. Another method of modification was the pillaring process; it consists of the intercalation of large inorganic molecules (mainly metal-hydroxy polycations) between the clay sheets in the first stage, followed by calcination in the temperature range of 300 to 500 °C, resulting to development of oxidic species between the smectite layers and an open up and rigid structure [25]. The resulting pillared clays exhibited good thermal stability at higher temperatures, higher surface areas, and higher acidity. The main application of the pillared clay minerals was in the catalysis filed; however, they were also used in environment remedial as adsorption of pollutants without or with additional modifications [26]. Since adsorption is a surface phenomenon, many studies were undertaken to improve the more and more the surface area of the pristine clay mineral and the removal sites in terms of hydroxyl groups, etc. The size of the pores played an important role in the requirement of the catalytic applications; however, in the case of the removal of organic dyes, the dyes were much larger and they could not access to these pores [27]. So the scientists came up with the idea to get larger pores and, at the same time, develop more exhibited surface areas greater than the pillared materials. These materials were called the porous heterostructures (known as PCHs). These materials were synthesized from swelling clay minerals, such as montmorillonite, and the clay layers were initially intercalated, using cationic surfactants via a cation exchange reaction, and later reacted with co-surfactant molecules (neutral aliphatic amines) and a silica precursor (such as Tetraethyl orthosilicate, TEOS) [28]. The hydrolysis and polymerization of silica species surrounding the template of the surfactant and co-surfactant occurred and produced a heterostructure template. After the removal of organic surfactants by calcination, a porous network of silica within the interlayer region was formed [29]. The incorporation of metal cations into the silica framework of PCHs induced additional acidic properties with the addition of the corresponding metal alkoxide (titanium or zirconium) with a silica source during the preparation process [30]. Another method consisted of the adsorption of the aluminum acetylacetonate complex followed by thermal decomposition, which resulted in aluminum oxide PCHs [31].

PCH materials are considered pillared clays with silica pillars inserted between the clay layers, with differences in the preparation and mechanism processes. Based on this idea, a novel synthesis method was proposed by [32,33], which consisted of integrating intercalated metal (aluminum or zirconium) species into clay minerals as precursors prior to reactions with C12 amine molecules and silica sources to prepare aluminum-incorporated PCH (Al-PCH) materials. These Al-PCH materials with fixed aluminum contents exhibited higher surface areas, mesoporous volumes, and Bronsted and Lewis acid sites [32,33]. This method has reduced the utilization of the organic template and, in one step, incorporated the aluminum or zirconium species precisely into the silica framework that was intercalated between the clay layers. Other metals were incorporated into the PCH structures and required different steps and consumed many chemicals and much time [34,35]. The type of the metal was selected based on the requirement of the catalytic reactions [36,37].

The PCHs have been used to adsorb anionic dyes, such as Remazol Violet 5R and Acid Blue 25, and metals [38,39], or as support for some metal oxides for the oxidation of methylene blue, and other catalytic reactions [37,40,41,42,43].

As mentioned above, few studies were concerned on the removal of basic dyes by using PCHs materials; in this study, PCH material with two different incorporated metals in the silica gallery was systemically undertaken to improve the microtextural properties of the parent clay and, thus, its efficiency as a removal agents for Basic Blue-41 (BB-41) dye. The Basic Blue-41 dye was selected because it has wide applications in different industrial sectors, and, especially in the textile industry, it is also applicable as a stainer for the recognition of avian leukocytes, blood, and bone-marrow cells [44,45]. The PCH precursors were prepared using an intercalated metal–clay to minimize the generation of organic waste, and to reduce the time of synthesis [32,33]. Thus, a reduction of the cost will be envisaged. The removal experiments of Basic Blue-41 were performed in a batch reactor, by controlling different parameters, such as the pH of the solution, initial concentrations, and the used amount of the solid. The reuse of the PCHs was examined after six cycles to elucidate their efficiency as suitable removal agent; this property was not reported in the literature for these kinds of materials. In this regard, a friendly method to the environment was applied based on the decomposition of the BB-41 on the surface of PCH materials, using oxone and cobalt solutions. The experimental data were fitted in different models to find the most suitable model in aim to propose a single batch reactor design for an eventual removal of BB-41 process, at a large scale.

## 2. Materials and Methods

### 2.1. Materials

Ca-montmoillonite (assigned as Ca-Mt) was ordered from the Source Clays repository, Purdue University (West Lafayette, IN, USA), with a cation exchange capacity (CEC) of 92 meq/100 g. Aluminum chlorhydrate hydroxide (Chlorhydrol, 50%) was provided by the Reheis Chemical Company, Berkeley Heights, NJ, USA. Zirconyl nitrate dehydrate (Zr(NO_3_)_4_. 2H_2_O), tetraethoxide orthosilicate (TEOS), and dodecylamine (C_12_ amine) surfactant were purchased from Aldrich, St. Louis, MO, USA. The chemicals were used as received.

Basic Blue-41 (from Aldrich) has the molecular formula C_20_H_26_N_4_O_6_S_2_ (mol. weight 482.57 g/mol) with a color index Number 11105.

### 2.2. Intercalated Clay Precursors

Aluminum-intercalated clay (Al-Mt) was prepared by dispersing a fixed amount of Ca-Mt in a solution of Chlorhydrol (50%) at a fixed value of Al mmol to clay of 6, as reported in our previous work [31]. A specific mass of Chlorhydrol solution was added to 200 mL of deionized water and aged at 80 °C, for 1 h, prior the addition of 5 g of Ca-Mt in dry-powder form. The suspension was further stirred for another 1 h at 80 °C; the suspension was cooled at room temperature, prior to filtration, and rigorously washed with deionized water and air-dried overnight.

Zr-intercalated precursor (Zr-Mt) was prepared by following the procedure reported above, by changing the Al solution by zirconium one and keeping the same ratio of Zr mmol to clay of 6, as reported in our previous work [32].

### 2.3. Preparation of Metal Porous Clay Heterostructures (Al-PCH and Zr-PCH)

The preparation was performed in two stages: the precursor was prepared, and then it was calcinated to remove the organic material. Thus, the term “precursor” describes the as-synthesized product (intercalated), and “material” defines the calcined precursors at 550 °C.

One gram of the aluminum-intercalated clay (Al-Mt, or Zr-Mt) was added separately to neutral amine (dodecylamine, C12 amine) and TEOS at molar ratios of clay/C_12_H_25_NH_2_/TEOS of approximately 1/20/150 [32,33]. The mixture was stirred at room temperature for a period of 4 h, using a magnetic stirrer. The obtained PCH precursors were recovered by filtration and air-dried overnight. The precursors were designated as Al-PCH and Zr-PCH precursors, respectively.

The Al-PCH and Zr-PCH materials were obtained after calcination of their corresponding precursors at 550 °C for 6 h in air at a heating rate of 1 °C/min, to eliminate the organic moieties of the C12 amine molecules.

### 2.4. BAsic Blue-41 Removal Procedure

The removal of BB-41 was studied in a batch mode, as reported previously for different solids. A fixed mass of 0.050 g from different samples was added to 50 mL of known concentrations (50–500 mg/L, step size: 50 mg/), in a series closed tubes. The used solutions were prepared by dilution from a stock solution (of 1000 mg/L). The tubes were shaken at 120 rpm, at 25 °C, for overnight. After centrifugation, the concentration at equilibrium of BB-41 solutions was analyzed, using a UV–Vis spectrophotometer (Agilent, Mulgrave, Australia).

The removal experiments were examined by changing the BB-41 C_i_, the pH of solution (by adding drops of NaOH (0.1 M) for basic media and HCl (0.1 M) for acidic media), and the amount of used solid (Al-PCH or Zr-PCH). If one parameter was changed, the other factors remained unchanged.

The regeneration study was performed by following the procedure described elsewhere. Briefly, fresh spent BB-41 saturated Al-PCH or Zr-PCH was treated with a solution of oxone containing Co^2+^ cations, for 30 min; the treated solids were recuperated by centrifugation and then treated again with a BB-41 solution with C_i_ value of 200 mg/L for 2 h. Then the same procedure was repeated after measuring the concentration at equilibrium (C_e_) for seven cycles.

### 2.5. Characterization

The X-ray diffraction (XRD) technique was used to follow up the structural changes during the two steps of the preparation of Al-PCH and Zr-PCH materials. The patterns were collected by using a Bruker diffractometer (Brucker, Karlsruhe, Germany) (model Advance 8) equipped with a Ni filter and Cu-K alpha radiation (λ = 1.5406 Å), in the range 2θ, from 1.5 to 50. The percentages of SiO_2_, Al_2_O_3_, and ZrO_2_ in Al-Mt and Zr-Mt and PCH precursors and materials were estimated, using X-ray fluorescence (XRF), from a Bruker Model S4. The thermogravimetric analysis (TGA) features were obtained by using the TA instrument (TA instruments, New Castle, DE, USA) model 9610 under atmospheric air and a heating rate of 10 mL/min, and heating rate of 5 °C/ min. The ^27^Al and ^29^Si solid MAS NMR spectra for the intercalated materials and related PCH materials were collected using a Bruker spectrometer as reported elsewhere and tetramethylsilane (TMS) as reference for the zero chemical shift. The FTIR spectra of the samples were collected by Biolab instrument (biolab, Ontario, Canada) in the range of 4000 to 400 cm^−1^ and using KBr pellet technique. The microtextural properties were estimated by using a Quantachrome Autosorb A6 machine (Quantachrome, Boynton Beach, FL, USA). The samples were cleaned under vacuum overnight at 150 °C. UV–Visible spectrophotometer (Mulgrave VIC, Australia) Varian (carry 100) was used to determine the concentrations of BB-41 at the absorbance at maximum wavelength (λ_max_ = 610 nm) and computed from the calibration curves.

## 3. Results and Discussion

### 3.1. X-ray Diffraction (XRD)

**Figure 1:**
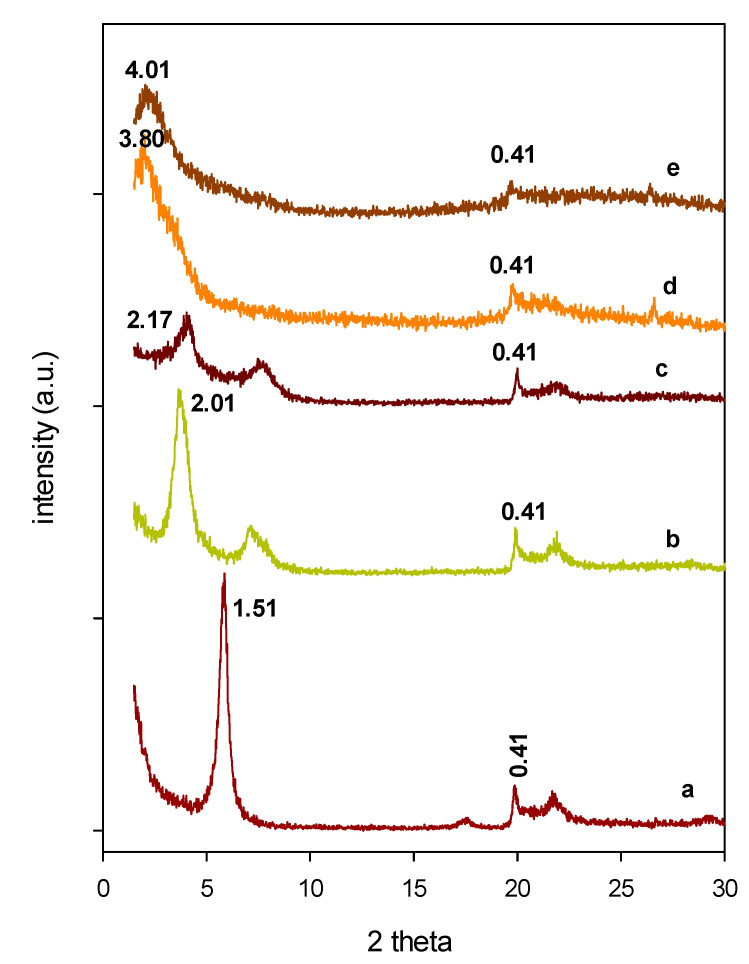
Powder XRD patterns of (**a**) starting Ca-Mt, (**b**) Al-Mt, (**c**) Zr-Mt, (**d**) Al-PCH, and (**e**) Z-PCH precursors.

The XRD patterns of the starting Ca-Mt and the intercalated precursors are depicted in Figure 1, along with the Al-PCH and Zr-PCH materials. The Ca-Mt pristine sample exhibited a basal spacing (d_001_) of 1.51 nm, and indicated the Ca cations were the major exchangeable cations, as previously reported [46]. After treatment with Chlorhydrol solution, the distance of the Al-intercalated clay (Al-Mt) between the clay layers expanded with an increase of the basal spacing to a value close of 2.09 nm. Taking into account the 0.96 nm thickness of the Mt layers, the Al-Mt precursor exhibited an interlayer spacing of 1.13 nm. This value was slightly similar to the size of Al_13_ polyoxocations, as reported in the literature [47]. A similar trend was observed when the raw clay was reacted with zirconyl nitrate solution: the zirconium intercalated clay (Zr-Mt) precursor exhibited a basal spacing of 2.17 nm, due to the presence of the Zr pillaring species [32]. In acidic solution, zirconium ions form tetrameric species with a chemical formula [Zr_4_(OH)_6_(H_2_O)_16_]^+^ and related dimensions of 0.89 nm × 0.89 nm × 0.58 nm [32]. The measured value of 2.17 nm corresponds to an interlayer spacing of 1.21 nm, as a result of the intercalation of zirconium polycations species with face-to-face stacked tetramers between the clay layers [32]. For Al-Mt and Zr-Mt precursors, the reflection peak at 0.41 nm that corresponded to the 110 reflection was observed, and it indicated that the layered structure was not severely altered during the exchange reaction.

After the reaction of Al-Mt with TEOS and C_12_-amine molecules, a further expansion of the basal distance was detected: the 001 reflection of Al-Mt shifted to lower angles and corresponded to a basal distance of 3.61 nm (Figure 1). A similar PXRD feature was obtained when Zr-Mt, treated with C_12_ amine and TEOS, with a new reflection at 4.01 nm. This value is substantially greater than that of the starting Zr-Mt precursor. In both cases, the expansion of the basal spacing indicated the co-intercalation of silica species and C12 surfactants between the clay layers, and the preparation of PCH precursors was successfully achieved [33]. However, when Ca-Mt (without prior modification) was reacted with pure C12 amine and TEOS, a slight expansion of basal spacing (d_001_) arose, from 1.51 to 1.72 nm, thus confirming the requirement to expand the interlayer spacing as a first stage prior the preparation of PCH precursors [33]. The obtained values of 3.61 and 4.01 nm were in the range reported for PCH precursors [32,33,48].

**Figure 2:**
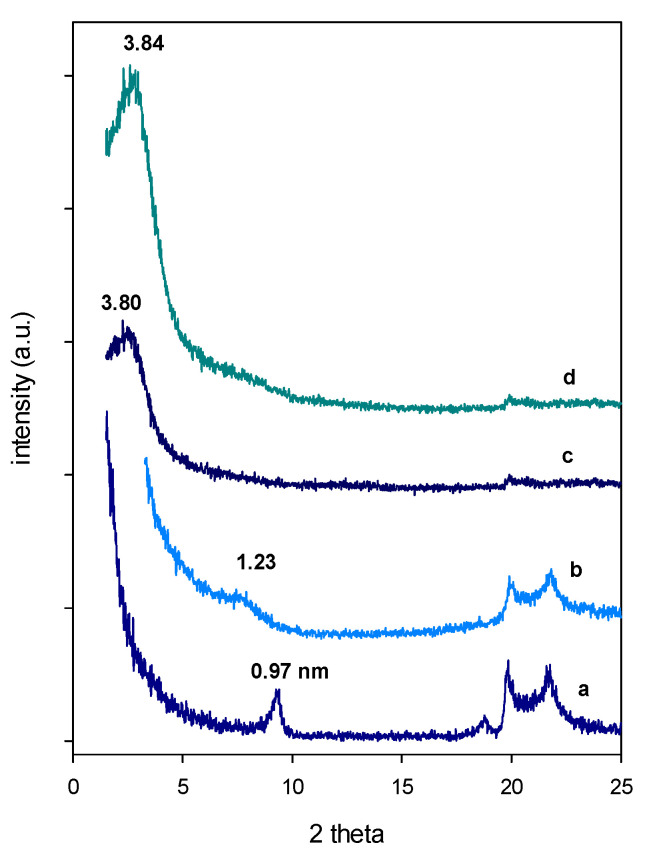
Powder XRD patterns of calcined (**a**) starting raw, (**b**) after reaction with C_12_ amine, and TEOS. (**c**) Al-PCH and (**d**) Zr-PCH after calcination at 550 °C.

After calcination at 550 °C of the Al-PCH and Zr-PCH precursors, the PXRD patterns indicated a reduction of the basal spacing to 3.80 and 3.84 nm, respectively (Figure 2). This fact resulted from the combustion of the organic moieties and to the formation and the condensation of three-dimensional silica species, as reported for PCH materials prepared using conventional methods [48,49].

The calcination of the precursor prepared from the raw Ca-Mt exhibited a basal spacing of 1.14 nm lower than the values reported for Al-PCH and Zr-PCH materials. This value (1.14 nm) was higher than the one reported for totally collapsed clay (of 0.96 nm) [46]. This slight variation was the consequence of silica species or organic materials present in the material (Figure 2).

**Figure 3:**
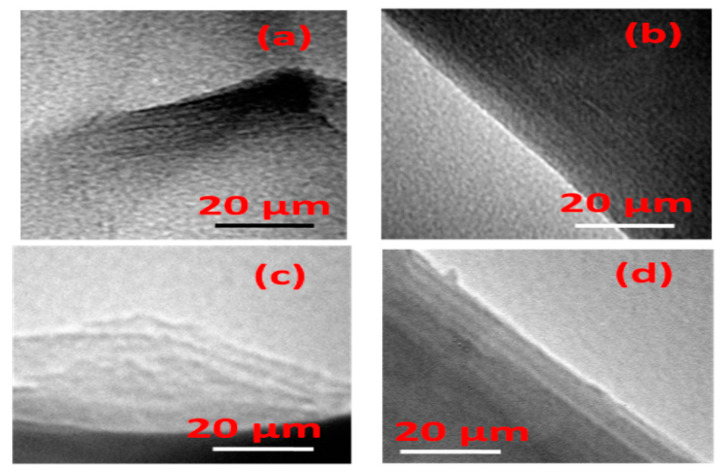
TEM micrographs of (**a**) Ca-Mt (**b**) after intercalation with Zr species (Zr-Mt). (**c**) Al-PCH and (**d**) Zr-PCH materials after calcination at 550 °C.

The TEM studies showed that the starting Mt clay was composed of stacked layers with curved edges (Figure 3a). Upon intercalation of aluminum or zirconium species, the layered feature of Al-Mt or Zr-Mt intercalated precursors was preserved, with certain expansion (Figure 3b). The basal spacing continued to swollen for Al-PCH and Zr-PCH materials (after reaction with C12 amine and TEOS) with a layered structure character (Figure 3c,d), as indicated by the XRD data.

### 3.2. XRF Data

The XRF analysis was executed to confirm the existence of Al, Zr, and Si species during the different steps of Al-PCH and Zr-PCH materials preparation. The reported data indicated that the intercalated of aluminum species was successfully achieved into the Ca-Mt raw clay with the reduction of CaO percentage into the obtained precursors. A decrease in SiO_2_ and other oxide contents was observed and can be related to an increase in Al_2_O_3_ oxide in the materials [33,47]. The chemical analysis indicated that the ratios of Al mmol/clay g were lower compared with the starting ratio of 6. Similar data were reported for other clay minerals using a same aluminum pillaring solution [50,51,52]. In the case of Zr-Mt, the Ca cations were also exchanged with Zr species, as a significant decrease in its contents was observed. The other percentages were reduced as the result of additional Zr amounts in the Zr-Mt. Possible leaching of some metals could be envisaged due to the acidic nature of the Zr solution [30,33].

In the case of the PCH materials (mean after calcination at 550 °C), a significant improvement of silica content was registered for both materials, due to the presence of silica network in the structure as proved by PXRD data; at the same time, the percentage of Al_2_O_3_ and ZrO_2_ decreased. This decrease could be related either to the exchange of the pillaring species by the C_12_ amine molecules or to the presence of significant amounts of SiO_2_ in the materials (Table 1). Indeed, in a separate experiment, when the Zr-Mt precursor was reacted with pure C_12_ amine molecules, the PXRD pattern indicated an increase of the basal spacing from 2.1 to 4.15 nm due to the presence of C_12_ amine molecules between the clay layers and a possible exchange of Zr species by the organic surfactants.

The percentages of Al and Zr in the PCH materials were in the same order in similar PCH ones prepared by various methods [37]. These data indicated the efficiency of the proposed method, by tuning the amounts of Al and Zr in the starting precursors.

### 3.3. FTIR Spectra

**Figure 4:**
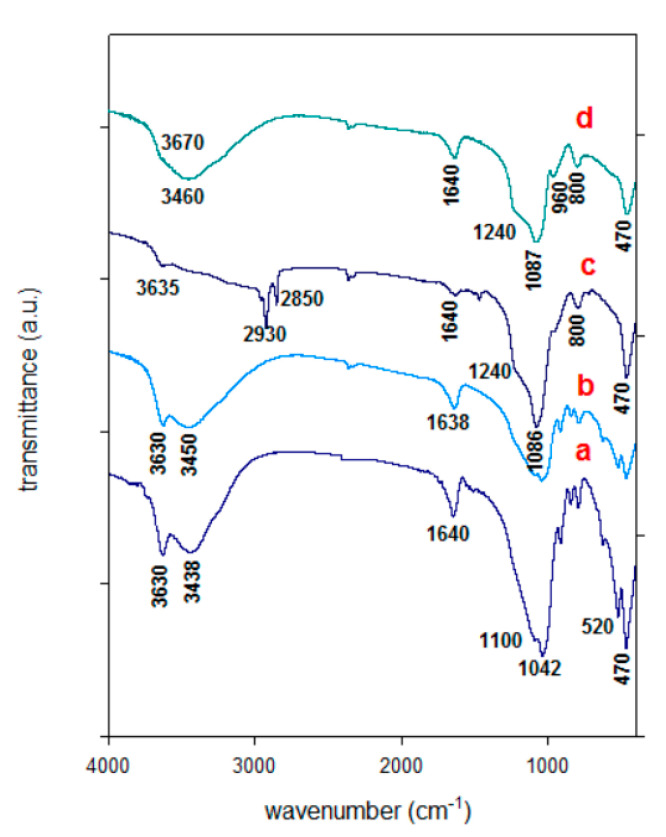
FTIR spectra of (**a**) starting Ca-Mt, (**b**) Al-Mt, (**c**) Al-PCH precursor; and (**d**) corresponds to (**c**) after calcination at 550 °C.

The FTIR spectra of the raw Ca-Mt and Al derived materials are presented in Figure 4. The assignment of the spectral bands was performed on the basis of published references. The Ca-Mt exhibited at 3630 cm^−1^ assigned to the stretching vibrations of the surface hydroxyl groups (metal–OH in the octahedral layer) and another one at 3438 cm^−1^ were related to adsorbed water in the interlayer, due to H-O-H stretching of water molecules forming a hydrogen bond in the interlayer region of Ca-Mt. The water molecules exhibited a band at 1640 cm^−1^. The intense band at 1042 cm^−1^ was related to the stretching vibration of Si-O groups; meanwhile, the bending vibrations of Al-O-Si and Si-O-Si were detected at 520 and 470 cm^−1^, respectively [21,53].

The Al-Mt and Zr-Mt precursors exhibited similar spectra to the one of starting Ca-Mt (for commodity, only Al-Mt spectrum was shown) and suggested that the structure of the parent clay was not altered through the intercalation of pillaring species, in good agreement with the PXRD data [32,33]. The intensity of the band at the 3447 and 1640 cm^−1^ regions increased in intensity, due to the presence of Al and Zr species and the hydroxyl groups involved in water–water hydrogen bonding. Some authors claimed that the intercalation process induced changes in the FTIR spectra, with increase of the intensity of some bands in the region of 700 to 500 cm^−1^; however, it was difficult to prove their claims, because no information in the characterization paragraph was reported on the preparation of their KBr pellets and the ratio of sample to KBr salt for each run.

The FTIR spectrum of Al-PCH precursor exhibited additional bands ranging from 3000 to 2800 cm^−1^, which agreed with the anti-symmetric and symmetric vibrations of methyl and methylene groups (–CH_2_) of the tails ‘s C_12_ amine surfactants [54], whereas a small band at the 1478 cm^−1^ region is ascribed to the bending vibrations of C–H [48,54]. (Figure 3). The detection of the bands related to water molecules indicated that not all the water was replaced by C12 amine and TEOS molecules. Overall, a decline in intensity in the region of 3700 to 3400 cm^−1^ was observed. The Al-PCH precursor exhibited additional intense bands at 1240 and 1084 cm^−1^ in comparison to Al-Mt and Zr-Mt; they were assigned to Si-O internal asymmetric stretching and associated with a three-dimensional silica network within the interlayer region. This is evidence of the presence of SiO_2_ species and the success of the presented method to prepare PCH materials [32,38]. Upon calcination at 550 °C of Al-PCH and Zr-PCH precursors, the organic moieties were completely removed by combustion in air, the related bands (in the range of 3000–2800 cm^−1^ and at 1478 cm^−1^) were completely vanished, and the shape of the Si-O bands were changed [34]. The spectrum was comparable to that reported for conventional PCH materials, with two intense bands at 1226 and 1077 cm^−1^ related to the formation of a SiO_2_-Al_2_O_3_ and/or SiO_2_-ZrO_2_ network within the PCH material; a new broad band at 4730 cm^−1^ was observed and assigned to the stretching vibration of silanol group (Si-OH) located between the two adjacent layers. More evidence of the presence of Zr-SiO_2_ and Al-SiO_2_ species were the bands located at 975–930 cm^−1^ and at 800 cm^−1^, as reported by Aguir et al. [38] (Figure 3).

### 3.4. TGA Analysis

**Figure 5:**
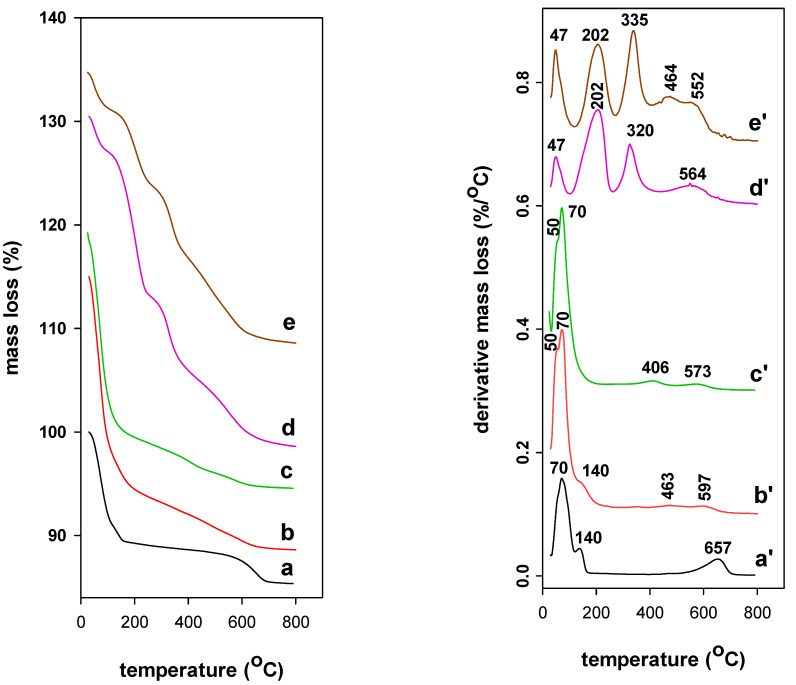
TGA and DTG features of (**a**,**a′**) starting Ca-Mt, (**b**,**b′**) Al-Mt, (**c**,**c′**) Zr-Mt, (**d**,**d′**) Al-PCH, and (**e**,**e′**) Z-PCH precursors.

Thermogravimetric features of different samples are shown in Figure 5 and were performed to verify the existence of pillaring species and surfactants in PCH precursors as well their thermal stability. As mentioned in a previous work [55], in some cases, it was hard to assess the separate temperature ranges for different mass losses. The derivative thermogravimetric (DTG) features were used as an alternative. They were computed by the apparatus software from TG data and represent dm/dT as a function of temperature. Ca-Mt raw clay exhibited a single mass of 12% from 25 to 200 °C, due to the desorption of physisorbed water on the external surface and to the dehydration of water molecules coordinated to Ca^2+^ cations, resulting in two peaks at 50 and 70 °C, respectively. A second mass loss of 4.5% in the range of 550 to 800 °C occurred and related to the dehydroxylation of the clay sheets with a maximum temperature loss of 657 °C [55].

After intercalation of Al species and Zr species, the TG features exhibited higher mass loss of 17% in the range of 25–230 °C, accompanied by two DTG peaks at 50 and 70 °C related to the loss of physisorbed water and the dehydration process in the interlayer spacing. Continuous mass loss of 4.5% occurred with a broad DTG peak centered at 573 °C (for Al-Mt) and (569 °C for Zr-Mt precursor), due to the dehydroxylation of the pillars entities and the clay sheets. In the case of Zr-Mt, dehydration of water molecules strongly attached to hydroxyl-zirconium cations and to the dehydroxylation of hydroxyl-zirconium species occurred continuously in the range of 200 to 500 °C, accompanied by a DTG peak at 406 °C. In overall, the total mass loss in the range of 25 to 800 °C, was higher than that of Ca-Mt, and reinforced the presence of Al and Zr species in the studied precursors [32,33].

After reaction with C_12_ amine and TEOS solutions, the Al-PCH and Zr-PCH precursors also exhibited similar DTG features, with a remarkable decrease of the mass lost step in the range of 25 to 120 °C from 17% to 4.5%, and accompanied with a significant reduction of the related peak’s intensity. This fact was due to the lower content of water and less numbers of hydroxyls on the surface, as a result of the presence of C_12_ amine molecules. The later was further confirmed by the presence of two DTG additional peaks with maximum temperatures values around 203 and 330 °C, and a mass loss of 20%. A continuous mass loss was observed from 400 °C, and associated to the complete removal of carbon residual materials and dehydroxylation process of Si species and clay sheets, associated with broad peak with a maximum temperature value of 564 °C for Al-PCH precursor and at 573 °C for Zr-PCH one. In overall, the Al-PCH and Zr-PCH precursors behave in a similar manner, and the TGA analysis did not indicate any difference of Al or Zr during the combustion and the dehydroxylation process, the overlapping of the peaks at higher temperatures have made difficult to note such effects [34,35].

### 3.5. Solid ^29^Si and ^27^Al NMR

The Solid NMR technique has proved its utility to differentiate between the different environments of Si and Al in the SiO_2_ network of the Al-PCH and Zr-PCH materials.

**Figure 6:**
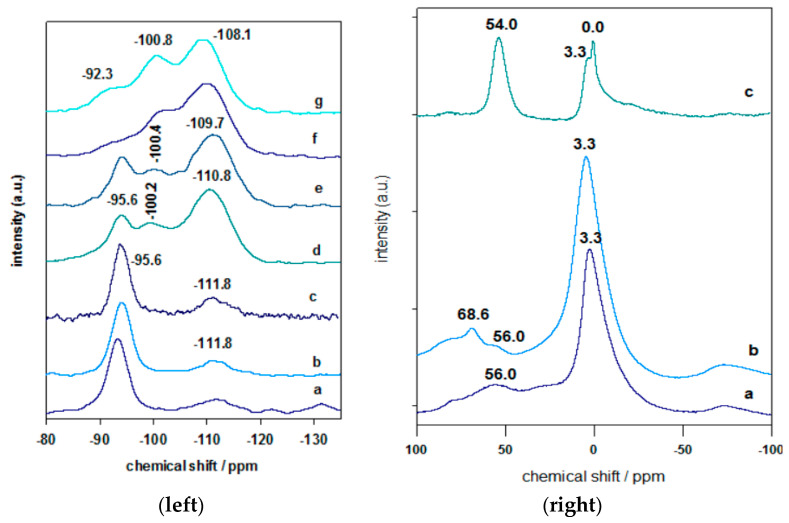
(**left**) Solid ^29^Si NMR spectra of (**a**) Ca-Mt starting material, (**b**) Al-Mt, and (**c**) Mt-Zr. (**d**) Al-PCH, and (**e**) Zr-PCH precursors. (**f**) and (**g**) correspond to Al-PCH and Zr-PCH after calcination at 550 °C. (**right**) Solid ^27^Al NMR spectra of (**a**) Ca-Mt starting material, (**b**) Al-Mt, and (**c**) Al-PCH material after calcination at 550 °C.

The ^29^Si MAS–NMR spectrum of Ca- Mt clay (Figure 6) displayed an intense resonance peak at −95.0 ppm, assigned to the Q^3^ Si atoms in the tetrahedral layer of the clay, linked through oxygen to three other Si atoms and either to an Al or Mg atom in the octahedral layer [56]. The second broad peak at −111.8 ppm was associated to silica impurities in the parent clay. Similar data was reported for other clay minerals. The Al-Mt and Zr-Mt precursors exhibited similar ^29^Si MAS–NMR spectra to the starting Ca-Mt, as reported in our previous works [32,33], with a slight shift of the main peak at −95.6 ppm; these data suggested that the overall structure of the clay mineral was not modified during the cation exchange reaction with Al or Zr species, with a preservation of basic structure of the clay sheets (Figure 6). Similar data were reported for other similar materials [32,33,57,58].

In the case of Al-PCH or Zr-PCH precursors, different features were obtained, with an intense resonance peak at −110.8 ppm and a weak one at −100.2 ppm (Figure 6). The characteristic resonance peak of TEOS at −81.7 ppm was not observed and confirmed the polycondensation of silicon species in the interlayer gallery of the obtained precursors [59]. The peaks at −110.8 and −100.2 ppm were assigned to Q_4_ Si species Si(SiO_4_) and Q_3_ Si species (HO)(SiO_3_), respectively, as reported in similar precursors prepared with different methods [37], in addition to the existing resonance at 94.1 ppm which was related to clay layers. The existence of Al or Zr in the precursors did not affect the position of the bands, due to the overlapping and broadness of these bands. When the precursors were calcined at 550 °C, the Q_4_ species represented the dominant compound in the ^29^Si MRN spectra, due to the dehydration/polycondensation of the silica species between the clay layers [59,60] (Figure 6). The resonance peak of the Q_4_ Si species became broadened and shifted −109.7 ppm. The Q_3_ mesoporous silica at −100.8 ppm was still detected. However, the Q_3_ Si band at −95.6 ppm (related to the clay layers) was decreased in intensity with a shift to −92.3 ppm, and it was embedded in the intense peaks of the silica species. These data indicated the structure of the Al-PCH and Zr-PCH was maintained with some transformation of the silica intercalated species [32,33].

**Figure 7:**
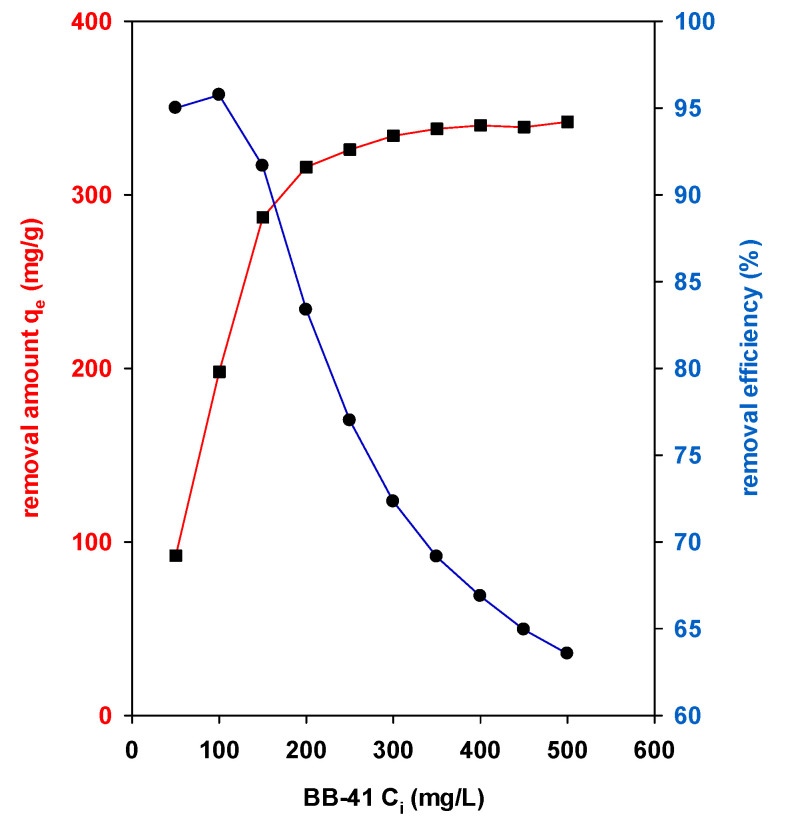
Effect of BB-41 initial concentration on the removal properties of Zr-PCH material (blue and red lines correspond to removal amount (mg/g) and removal percentage (%), respectively).

The existence of aluminum species in Al-Mt intercalated precursor was confirmed by the solid NMR technique (Figure 7). The ^27^Al MAS NMR spectrum exhibited an intense peak at 3.3 ppm, corresponding to Al in an octahedral environment (Al^VI^), and 56.0 ppm, related to tetrahedrally coordinated Al (Al^IV^) of the clay layers [56]. The peak situated between 60 and 70 ppm was resulted from the tetrahedral Al from the pillaring species [47]. However, in the Al-PCH material (calcined at 550 °C), a different feature was observed, with a substantial enhancement of the resonance at 54.0 ppm correlated to tetrahedral Al^IV^ and two resonance peaks associated to Al^VI^ at 3.3 and 0.0 ppm. These two peaks could be ascribed to the Al^VI^ in the clay sheets and an extra Al^IV^ existing in different environments, within the intercalated silica species [32,33].

### 3.6. Microtextural Properties

The microtextural properties of Ca-Mt, intercalated precursors (Al-Mt and Zr-Mt) and their PCH derivatives are presented in Table 2. The starting Ca-Mt displayed a S_BET_ value of 90 m^2^/g. This value is above the values reported for other raw clay materials. However, Al-Mt and Zr-Mt precursors exhibited a higher S_BET_ of 281 and 277 m^2^/g, respectively. The improvement of the S_BET_ indicated the successful intercalation of pillaring species. The presence of the pillaring species induced certain microporosity (of 0.024 and 0.088 cm^3^/g) in addition to some mesoporosity [32,33]. The Al-Mt precursor exhibited a total pore volume of 0.257 cm^3^/g higher than the Zr-Mt (0.324 cm^3^/g). The average pore diameter values indicated the mesoporous character of the starting intercalated precursors (see Table 2).

After reaction with C_12_ amine molecules and TEOS, the S_BET_ values of Al-PCH and Zr-PCH materials (after calcination at 550 °C) were improved significantly with values of 880and 915 m^2^/g, respectively. The micropore volumes decreased significantly, and the mesopore volume values were enhanced to 0.861 and 0.801 cm^3^/g. The materials retained the mesoporous character with an increase of the average pore diameter values of 3.82 and 4.67 nm. The microtextural properties of the studied PCH materials were higher compared to those reported for PCH materials prepared by conventional manners reported in the literature [48,60,61], and the proposed method could be used to tune the properties of the obtained PCHs; indeed, previous studies have demonstrated that these characteristics were altered by varying the ratios of Al mmoles to clay or Zr mmoles to clay prior the preparation of the PCH derivatives.

## 4. Operational Effect on the Removal Properties of Basic Blue-41

### 4.1. Effect of BB-41 Initial Concentrations

The equilibrium experiments were carried out at least for overnight to insure the equilibrium state was attained. A series of equilibrium removal tests for BB-41 dye were performed at different initial concentrations (C_i_) from 50 to 500 mg/L. Figure 7 depicts variation of the removal efficiency (R%) and amount (q_e_ in mg/g) of Zr-PCH material. The removal capacity of Zr-PCH was increased significantly at the initial stage, from 50 to 250 mg/g, with an increase of BB-41 C_i_ values from 25 to 200 mg/L. Meanwhile, the removal efficiency (R%) declined. These data indicate that the driving force for the removal of BB-41 onto Zr-PCH was mainly the concentration gradient of BB-41 molecules [62,63]. When the removal sites of Zr-PCH attained a saturation, no further BB-41 molecules were removed, even under a larger adsorption force by further increasing of Ci values. Thus, the removed amount maintained as nearly unchanged for C_i_ of BB-41 concentrations exceeding 300 mg/L, and the removal efficiency continued to decrease [64,65]. The Al-PCH material showed the same trend, with a decrease of the reported values.

### 4.2. Effect of Zr-PCH Dose

**Figure 8:**
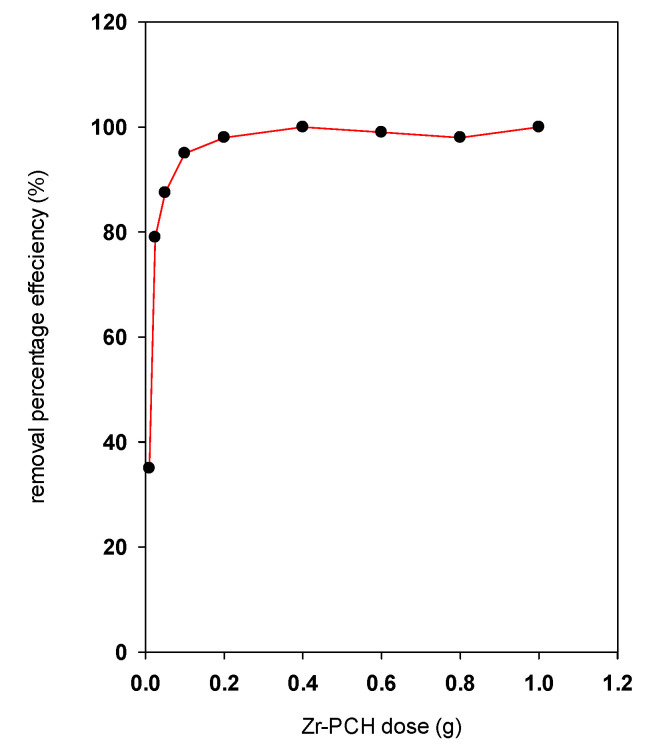
Effect of Zr-PCH amount (g) on the removal percentage of BB-41.

Different experiments were performed to study the effect of the used mass of Zr-PCH on the removal of BB-41, at a fixed C_i_ of a value of 200 mg/L and volume of 50 mL. Figure 8 depicts the effect of the added Zr-PCH mass to a volume of 50 mL of BB-41 solution, and the results showed that, in general, the removal percentage of BB-41 increased with Zr-PCH mass, owing to the increase in total available surface area and the number of removal sites on the Zr-PCH surface which allowed more bindings of BB-41 molecules. However, further addition of Zr-PCH mass did not significantly vary the BB-41 removal percentage, and a value of 99% was obtained for values higher than 0.4 g. Similar data were reported for Al-PCH material, and for other different solids using different dyes [63,64,65,66].

### 4.3. Effect of pH

**Figure 9:**
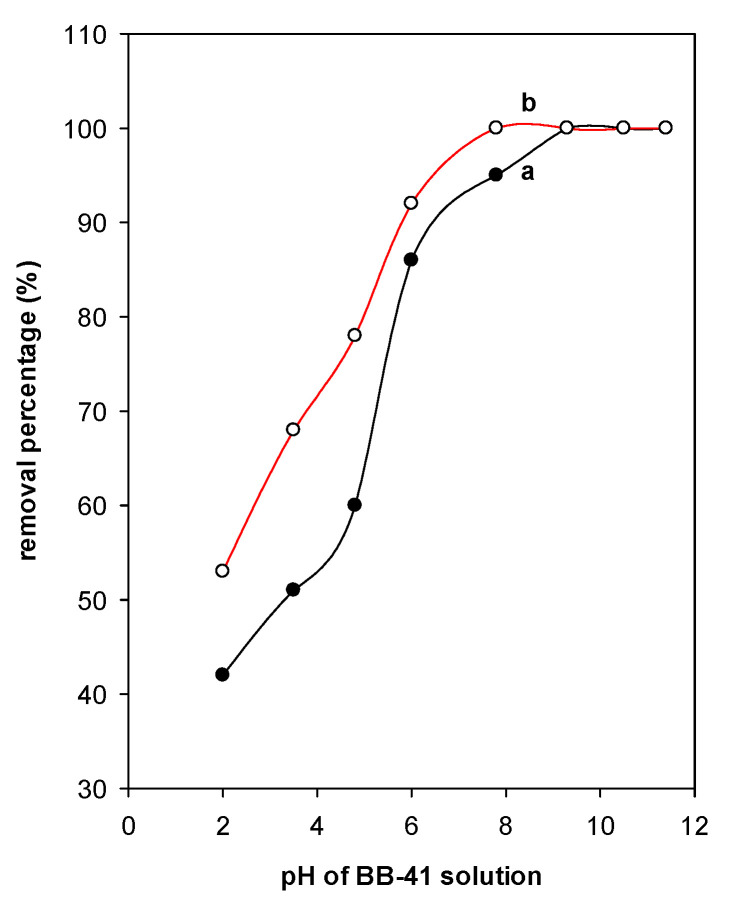
Effect of pH on the removal percentage (%) of (**a**) Al-PCH and (**b**) Zr-PCH materials.

The BB-41 ionizes in solution, once it dissolved in water, becoming positively ordered component, and releases cations in solution. Thus, the removal of BB-41 will be affected by both the charges of PCH materials and the dye molecules. Figure 9 illustrates the variation of removal percentage of Al-PCH and Zr-PCH materials with the initial pH of BB-41 solution. In general, the removal percentage increased with the increase of pH up to 6; then it reached a maximum of 100% at pH values between 6 to 9. At pH values higher than 9, the BB-41 molecules decomposed in the strong alkaline environment and formed a brown precipitate. Thus, we could not carry out the analysis of supernatant [64,67].

The decrease of removal efficiency at lower pH values was a result of the presence of species with the same charge—the dye molecules and the surface of PCH materials—or to the competition between the excess protons ions and the cationic dye groups for the removal sites. The point of zero charge pH (pHpzc) is another important feature that determines the pH at which the adsorbent surface has net electrical neutrality. At pH < pHpzc, the functional groups are protonated and a positive surface charge dominates, whereas they have a net positive charge when the equilibrium pH in the solution is lower than their pH_PZC_. The point zero charge of the starting Ca-Mt found to be in the range between 8 and 9.5 and close to 7.5 [40]. However, for Zr-Mt and Al-Mt precursors, the pH_PZC_ are shifted to acidic pH to values of 6.2 to 4.9 and 4.1 to 4.7, respectively [68,69], related to the property of the polycations utilized in the pillaring process. The pH_PZC_ values of Zr pillared clays are more acidic compared to Al pillared samples [69,70]. For the PCH materials, the reported values of pH_pzc_ were in the range of 3 to 4 [70], a deprotonation of the silanol groups (Si-OH) in the silicon oxide pillars intercalated during the preparation of the PCH; and negative surface charge sites increased. Indeed, the presence of these groups has been demonstrated also on the surface of MCM material and in the silica (SiO_2_) [71,72]. It is evident that electrostatic interaction was the main mechanism and promote the BB-41 removal process. Another mode of adsorption (ion exchange) could be envisaged, as the results are in agreement with the literature report [73].

### 4.4. Isotherms of Adsorption

To relate the experimental data to the theoretical predictions, two models were selected because they were widely applied to study the removal mechanism of the dye removals. [74]. For example, the Langmuir isotherm model presumes that the removal takes place at specific homogeneous sites on the adsorbent surface, and the adsorption is a monolayer. This model is applied to estimate the maximum capacity removal of the adsorbent [75].

The linear equation of the Langmuir model is expressed in Equation (1):

(1) $$\frac{C_{e}}{q_{e}}=\frac{1}{q_{max}\cdot K_{L}}+\frac{C_{e}}{q_{max}}$$

where *q_e_* and *C_e_* are the removed amount of dye (mg/g) and the concentration (mg /L) in the solution at equilibrium, respectively; *q_max_* is the maximum removed amount (mg/g), and *K_L_* (L/mg) is the Langmuir constant. The linear plot of *C_e_/q_e_* vs. Ce was used to evaluate these constants.

Contrary to the Langmuir model, the Freundlich isotherm model describes the experimental data for heterogeneous surfaces and non-uniform distribution of adsorption heat [76], as well as multilayer sorption.

The Freundlich model with the linear equation is presented in the Equation (2):

(2) $$Lnq_{e}=LnK_{F}+\;LnC_{e}$$

where the *Ln* of the removed amount (*q_e_*, mg/g) is linearly related to the *Ln* of BB-41 dye concentration at equilibrium (*C_e_*), and *K_F_* and 1/n are the Freundlich constants. *K_F_* is a combined measure of both the adsorption capacity and affinity, and 1/n indicates the degree or intensity of the removed BB-41 dye.

The calculated parameters from the two models and the regression coefficients (R^2^) are presented in Table 3. The Freundlich model predicts that the dye concentrations on the adsorbent will increase so long as there is an increase in the dye concentration in the liquid. However, the experimental adsorption isotherm for BB-41 presented a long plateau at higher equilibrium concentrations, implying the presence of a monolayer; thus, the Freundlich model was not adequate to this study by considering the R^2^ value (0.7635). However, the R^2^ value was close to 1 for the Langmuir model and indicated that, overall, this model was the best model to describe the removal of BB-41 onto PCH materials, indicating monolayer coverage of the surfaces. 

The Ca-Mt raw material exhibited a maximum removal capacity of 60 mg/g; this value was close to that reported for similar materials [23]. When the raw clay was intercalated with Al or Zr species, the maximum removal capacities were also improved due to the accessibility of the removal sites after expansion by the pillaring species. However, they were still lower than those of the corresponding PCH materials. In other studies, the significant improvement in the dye removal efficiency of cannot be totally ascribed to the expansion of the interlayer space, and the catalytic degradation of the dye may be involved [77]. Similar results of the high removal efficiency of cationic dyes by pillared bentonites were recorded [70,78].

Table 4 summarizes a compilation of some used materials for the removal of Basic Blue-41, mainly aluminosilicates are presented, of course other bio-adsorbents were reported, but they were not reported in the present study. The data indicated that the PCH materials have a noticeable removal amounts in comparison with other intercalated clays and other aluminosilicates. The difference could be related to mesoporous character of the PCH materials, and the higher surface areas exhibited by these materials. The Al-PCH and Zr-PCH materials have a potential application in wastewater treatment for removing some environmental contaminants.

### 4.5. Theoretical Calculation

The adsorption is a surface impact, and it can be categorized into physical or chemical adsorption, depending on the different modes of interaction between adsorbent and adsorbate [82]. The main interactions are the Van der Waals’ forces between the adsorbent and adsorbate in the physical adsorption because there are no chemical bonds created between the adsorbent and adsorbates. The potential forms of physical adsorption would be hydrogen bonds and electrostatic effect. The main causes that modified the physical adsorption are the electrostatic effect, the electric intensity of hydrogen bond, the steric hindrance intensity of adsorbate, etc. [83].

In this study, the possible interaction sites through computation of electronic charge distribution and electrostatic potential was investigated. The interactions between silanols groups of PCH materials and BB-41 dye were considered.

**Figure 10:**
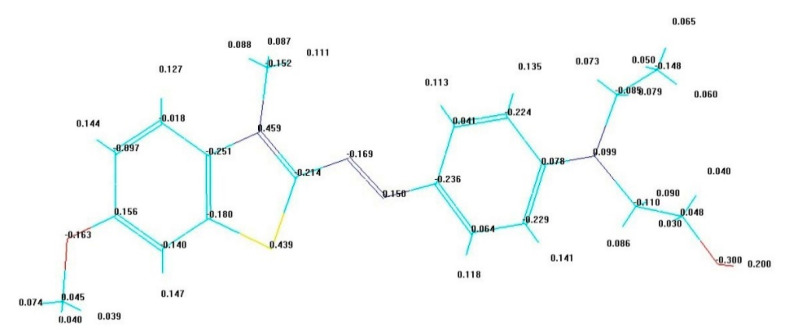
Mulliken atomic charges distribution (e^−^) of Basic Blue-41 molecule, as deduced from their optimized PM3 molecular geometries.

Mulliken atomic charges calculated by the semi-empirical PM3 theoretical method are presented in Figure 10. It shows that, for BB-41dye, the most of the negative charges were centered onto the oxygen atoms and some nitrogen atoms, while the positive charges were situated on other nitrogen and sulfur atoms. In other words, the half side of the molecule with sulfur and nitrogen heteroatom’s is more positively charged. It should also be noted that the positive charge on the hydrogen atom of the hydroxyl group (OH) in the second half of the molecule has a very marked positive charge (0.2 e^−^).

**Figure 11:**
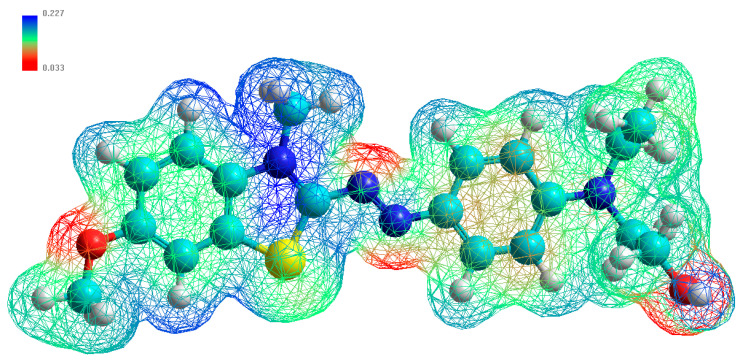
Three-dimensional (3D) mapped isosurface of the electrostatic potential surrounding BB-41 molecule.

The electrostatic potential (ESP) is important for understanding the chemical reactivity and the atomic structure of molecules and solids [84]. It is useful for finding sites of interaction in a molecule: negatively charged species which tend to attack the sites where the electrostatic potential is strongly positive. Figure 11 illustrates a three-dimensional (3D) mapped isosurface of the electrostatic potential surrounding BB-41 molecule. The red colors indicate weak positive ESP regions, followed by green colors, which denote slightly positive ESP regions, and blue colors, indicating strong positive ESP regions.

In the case of electrostatic attraction with a negatively charged surface like the surface of PCH, the BB-41 molecules will have a tendency to orient themselves preferentially to the surface by the sides possessing the highest values of positive charge density. The orientation via the hydrogen of alkyl amine group -NCH_3_ is situated in the first half of the molecule or to the orientation with a cycle having the suffer (S) atom, and finally, there is orientation with the hydrogen of hydroxyl group of the second side of the molecule. One could envisage the orientation positions of BB-41 are parallel with respect to the negatively charged surface of the clay layers, as presented in Figure 10. Indeed, as reported, the removal of BB-41 by montmorillonite clay occurred on the outer surface of the clay sheets at lower initial concentrations, and then an intercalation of these dyes happened at higher C_i_ values [67]. The BB-41 adopted a parallel orientation to the clay layers inside the interlayer space of the clays and formed one monolayer of 0.48 nm and then a bilayer of 0.96 nm [67]. This value was close to that reported for the BB-41 dimensions [66].

In addition to surface adsorption, the BB-41 molecules can take place through diffusion in the pore structures of PCH materials, lengthwise; this mechanism was hypothesized for some materials [85,86].

### 4.6. Regeneration Properties

The regeneration process is an important factor for the feasibility of the removal process, and it will add value to the used materials.

Recycling the spent adsorbents is counted as an important economic aspect in reducing the process expenses. Different methods were reported to regenerate the adsorbents after dye removals [87,88]. As a sustainable development approach, the regeneration study was limited only to a friendly environment method in order not to produce hazardous materials. It consists of treating the fresh material after the removal process with a solution of oxone and cobalt solution. The oxone and cobalt solution was employed so many times, without changing it each time, thus it will reduce the waste amount of cobalt solution and its effect on the environment [64].

**Figure 12:**
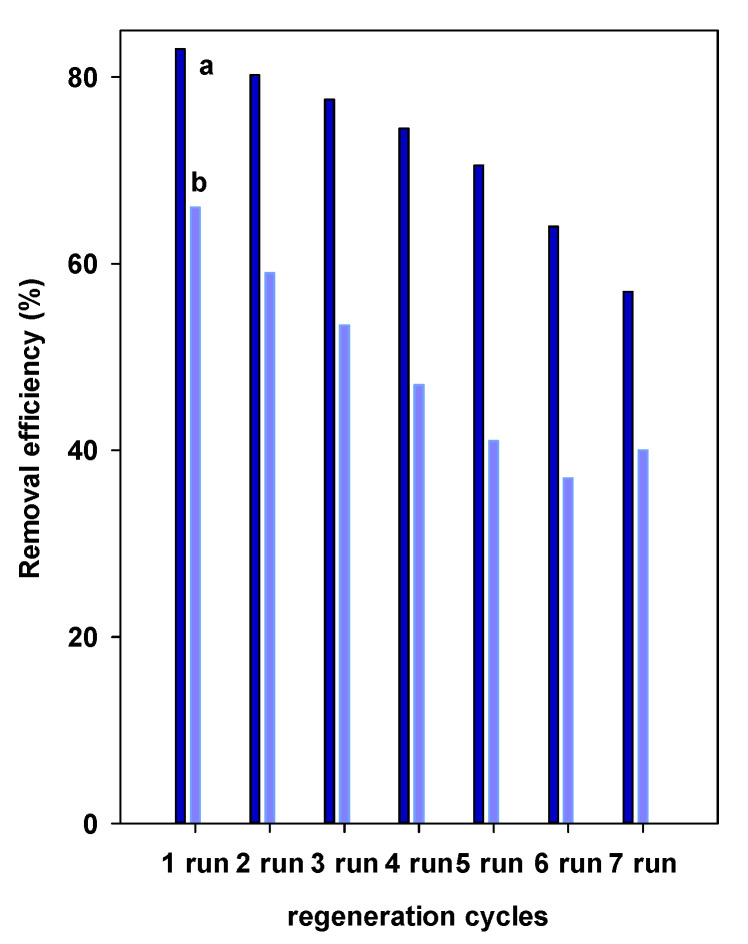
Regeneration properties of (**a**) Zr-PCH and (**b**) Al-PCH materials.

Figure 12 presents the experimental results of the removal efficiency (*R*%) in the first six removal–regeneration cycles for Al-PCH and Zr-PCH materials. The Zr-PCH material exhibited sustainable removal properties after five cycles of reuse, with a decrease of the removal percentage by 15%, while, in the case of Al-PCH material, the investigation indicated that the removal properties were maintained up to three cycles, with a decrease of the initial value of 66.5% to 53%; this value continued to drop after six runs, to a value of 45%. This later value was not achieved for Zr-PCH material even after six tests cycles.

These data indicated that the destruction of removed BB-41 dyes was easily achieved for Zr-PCH materials compared to Al-PCH, and indicated that the removed BB-41 molecules were strongly attached to the removal sites of Al-PCH compared to Zr-PCHs; another possible reason is that the destruction of dyes molecules by this method was improved in the case of Zr-PCH, and it could be related to the acidity of the PCH materials. Indeed, the Zr-PCH material exhibited higher acidity compared to Al-PCH as measured by cyclohexylamine desorption method [32,33]. Overall, the effectiveness of the proposed method for regeneration makes it a promising method and indicates that the Zr-PCH material has a strong regeneration performance and can be reused as a good material to efficiently remove BB-41 at least five times.

### 4.7. Single-Stage Batch Design Process

To scale up the outcomes of the laboratory scale results, a single batch adsorber was evaluated as an approach for design analysis. This can be used to predict the required amount of materials necessary to remove a certain percentage of the pollutant from specified volumes of contaminated water [89,90].

The design objective was to predict the required amount of adsorbents necessary to reduce the concentration of BB-41 dye from *C_o_* (mg/L) to *C_1_* (mg/L) of wastewater volume (L) and to adsorbent is M (g). [65,89,90], The removed BB-41 (mg dye per g of solid) varies from *q_o_* (mg/g) to *q_1_* (mg/g). The mass balance at equilibrium condition is given as follows:

(3) $$V\left(C_{o}-C_{e}\right)=m\left(q_{o}-q_{e}\right)=mq_{e}$$

In the present case, the removal of BB-41 fit well with the Langmuir isotherm. As a result, the parameters of Langmuir equation can be used effectively to achieve the proposed design, and the rearranged form is presented in Equation (4):

(4) $$\frac{m}{V}=\frac{C_{o}-C_{e}}{q_{e}}=\frac{C_{o}-C_{e}}{\frac{q_{m}K_{L}C_{e}}{1+K_{L}C_{e}}}$$

By replacing *C_o_ − C_e_* and *C_e_* expressions by *C_o_* and removal percentage (*R*%), the Equation (4) could be rewritten as follows [91]:

(5) $$\frac{m}{V}=\frac{C_{o}-C_{e}}{q_{e}}=\frac{RC_{o}}{q_{m}\frac{K_{L}\left(1-R\right)C_{o}}{1+K_{L}\left(1-R\right)C_{o}}}$$

**Figure 13:**
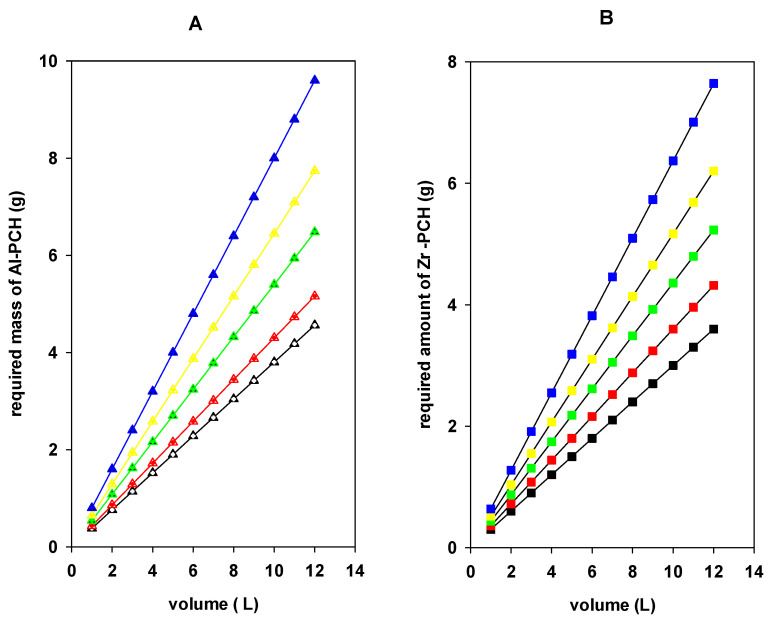
Required mass of (**A**) Al-PCH and (**B**) Zr-PCH to treat different volumes of BB-41 solutions.

Figure 13 illustrate series of plots (obtained from Equation (5) to predict the required amounts of Al-PCH and Zr-PCH materials, necessary to reduce the initial concentration of BB-41 dye (200 mg/L) to final concentrations of 100, 80, 60, 40, and 10 mg/L, using different volumes of BB-41 in the range of 1 to 12 L, in 1 L increments. In general, the needed mass of PCH materials increased as the target percentage increased, from 50%, 60%, 70%, 80% and 90% removal of BB-41 from 10 L contaminated solution. For example, 3.80, 4.30, 5.41, 6.45, and 8.01 g of Al-PCH materials are required, respectively. However, when Zr-PCH materials were utilized, the required amounts were 3.0, 3.60, 4.36, 5.17, and 6.37 g for the same removal percentages. These values were less than those of Al-PCHs (about 20%) due to the higher removal capacity of the Zr-PCH materials, as reported in Table 3.

## 5. Conclusions

The proposed method to prepare porous clay heterostructures from intercalated polyoxocations aluminum or zirconium led to reduce the amount of organic surfactants and to the insertion of Al or Zr cations into the silica moiety in a single step. The obtained materials have higher surface-area properties compared to those prepared by the conventional method, and they also have good thermal stability. The presence of the intercalated species was essential to obtain such materials. The Al-PCH and Zr-PCH materials exhibited higher surfaces areas of 770 and 915 m^2^/g, respectively, with an average pore diameter larger than the diameter of BB-41 molecule. The removal of BB-41 dye from artificially contaminated water was tested in this study, and the removal efficiency was related mainly to initial concentration values, the mass of the used material, and the pH value of the starting BB-41 solution. The experimental data fitted the Langmuir model with a formation of a monolayer, and the deduced maximum removal capacities indicated that PCH materials exhibited higher removal amounts compared to the starting Ca-Mt, with values of 323 and 274 mg/g for Zr-PCH and Al-PCH, respectively. The regeneration tests indicated that the Zr-PCH has a stable reuse for five runs, which is not the case of Al-PCH; this difference could be related to the high acidity of Zr-PCH compared to Al-PCH that helped the destruction of the removed BB-41 during the reaction with oxone and cobalt cations. A single-stage batch design was proposed, using the Langmuir model and the mass balance equation; an increase of the removal capacity of Zr-PCH led to the reduction of the predicted amounts for the target removal of BB-41 dye of 20 to 30%. In overall, the prepared PCH materials were proposed as an alternative removal agent of BB-41.

## Figures and Tables

**Table 1:** Chemical analysis of some oxides (in mass %) of Ca-Mt, Al-Mt, Zr-Mt, and their PCH derivatives.

Samples	SiO_2_	Al_2_O_3_	MgO	Fe_2_O_3_	CaO	TiO_2_	K_2_O	MO *
Ca-Mt	72.2	14.5	3.09	0.884	1.77	0.236	0.108	0.00
Al-Mt	55.0	20.2	2.43	0.489	0.05	0.019	0.062	12.01
Al-PCH	80.1	6.43	0.61	0.141	0.02	0.032	0.030	10.26
Zr-Mt	49.10	8.65	2.27	0.663	0.04	0.021	0.062	11.00
Zr-PCH	79.9	5.54	0.66	0.130	0.014	0.002	0.030	7.83

MO * corresponds to Al_2_O_3_ in the case of Al-Mt and Al-PCH; ZrO_2_ for Zr-Mt and Zr-PCH.

**Table 2:** Specific surface areas (S_BET_), total pore volume (TPV) and average pore diameter (APD) of the starting clay and derived materials.

Samples	S_BET_ (m^2^/g)	µpore Volume *	TPV (cc/g)	APD (nm)
Ca-Mt	90	0.00	0.051	5.81
Al-Mt	281	0.083	0.262	3.66
Al-PCH	850	0.00	0.851	3.82
Zr-Mt	277	0.125	0.224	2.62
Zr-PCH	918	0.082	0.801	3.24

* µpore volume (micropore volume m^2^/g).

**Table 3:** Estimated Langmuir and Freundlich parameters for the studied materials.

Samples	*q_m_* (mg/g)	*K_L_*	R^2^	*K_F_*	1/n	R^2^
Ca-Mt	57	0.0289	0.9854	5.44	0.5203	0.7120
Al-Mt	88	0.0564	0.9986	7.53	0.412	0.7531
Al-PCH	274	0.2280	0.9996	63.34	0.37	0.7635
Zr-Mt	114	0.07720	0.9991	13.68	0.433	0.7641
Zr-PCH	346	0.2122	0.9996	188.85	0.15	0.7603

**Table 4:** Removal properties of selected materials.

Materials	*q_m_* (mg/g)	Reference
Saudi Local clay	50–70	[23]
Natural zeolite	77	[66]
Sodalite zeolite	39	[79]
Zeolite X	27	[80]
Al-Pillared clays	88	This study
Zr- Pillared clays	114	This study
Al-PCH material	274	This study
Zr-PCH material	346	This study
Brick waste materials	60–70	[64]
nanoporous silica	345	[81]

## Data Availability

This study did not report any data.
